# Aorta Remodeling Responses to Distinct Atherogenic Stimuli: Hyperten-sion, Hypercholesterolemia and Turbulent Flow/Low Wall Shear Stress

**DOI:** 10.2174/1874192400802010041

**Published:** 2008-06-13

**Authors:** Cibele M Prado, Marcos A Rossi

**Affiliations:** Department of Pathology, Faculty of Medicine of Ribeirão Preto, University of São Paulo, S.P., Brazil

## Abstract

This review is based on recently published data from our laboratory. We investigated the role of hypertension and laminar flow, hypercholesterolemia and laminar flow and turbulent blood flow/low wall shear stress, and turbulent blood flow/low wall shear stress associated with hypercholesterolemia on aorta remodeling of rats feeding normal diet or hypercholesterolemic diet. Our findings suggest that increased circumferential wall tension due to hypertension plays a key role in the remodeling through biomechanical effects on oxidative stress and increased TGF-β expression; the remodeling observed in the presence of hypercholesterolemia could be initiated by oxidative stress that is involved in several processes of atherogenesis and this remodeling is more pronounced in the presence of turbulent blood flow/low wall shear stress.

## INTRODUCTION

Atherosclerosis is a systemic, diffuse process with multi-focal distribution involving the aorta and its conduits and by which the vascular intima becomes thickened with lipid rich gruel (atheroma) and connective tissue (sclerosis). The atherosclerotic lesions localize preferentially in particular regions of the arterial wall, like bends, branches and bifurcations [1,2]. Among known risk factors, hypertension and hypercholesterolemia are considered of utmost importance. Hypertension causes intimal and medial thickening and increase in connective tissue content [3]. The exact underlying mechanism of the association between hypertension and atherosclerosis is, however, not fully understood [4]. The association between cholesterol levels and atherogenesis was for the first time experimentally demonstrated in rabbits almost a century ago [5]. The twentieth century was the era of cholesterol and lipoproteins, culminating in a series of large-scale clinical trials showing conclusively that correcting hypercholesterolemia profoundly reduces morbidity and mortality from disease of the coronary arteries of the heart [6]. Recent serial clinical and intravascular ultrasound trials have demonstrated concordant beneficial effects of lipid-lowering treatment on arterial remodeling, including a reduction in adverse cardiovascular events and atherosclerotic plaque stabilization [7-9]. Taking into account the multi-focal distribution of the atherosclerotic plaques, it is a challenge to explain how equal concentrations of cholesterol bathing the endothelium or high blood pressure levels can produce local rather than global effects on arteries. Since there are numerous reproducible sites that are prone to developing atherosclerosis, a localizing element should be operating.

The high-susceptibility sites prone to development of atherosclerosis are thought to be conditioned by hemodynamic parameters, particularly associated with regions of low wall shear stress, oscillatory flow, or turbulent flow [2, 10, 11]. Although they are not by themselves responsible for the pathogenesis of atherosclerosis, they may prime the local vascular wall in which the lesions develop. Wall shear stress and stretch are the most important hemodynamic forces involved [12-14]. Shear stress is a frictional force parallel to the wall at the surface of the endothelium. Low wall shear stress, especially when blood flow is turbulent, is said to play important role in the pathogenesis of the atherosclerotic plaque [13]. On the other hand, the stretch stimulus can be evaluated as two factors: circumferential wall tension and tensile stress. The first one is due to transmural pressure and the second one act perpendicularly to the arterial wall and results from the dilating effect of blood pressure on the vessel. High stretch stimulus also plays a key role in the progress of atherosclerosis [12-15]. Designing experiments that allow the establishment of a hydrodynamic milieu to study how hemodynamic forces interplay with risk factors appears to be a very useful strategy. This review is based on recently published data from our laboratory analyzing the role of hypertension, hypercholesterolemia and turbulent blood flow on aorta remodeling of rats feeding normal or hypercholesterolemic diet.

## AORTA COARCTATION MODEL AND BLOOD PRESSURE ANALYSIS

The abdominal aorta was narrowed just below the diaphragm as described previously [16]; sham-operated animals underwent an identical surgical procedure, but aortic constriction was omitted. The constriction reduced about 80% of the aorta lumen. Animals were divided in sham-operated, operated, sham-operated+hypercholesterolemic diet (HD), and operated+HD. The aortic tube proximal (prestenotic segment) and distal (poststenotic segment) to the stenosis or corresponding segments from sham-operated animals were transversally cut into 5-6 mm long fragments.

This model of aortic constriction produces a hypertensive prestenotic segment associated with increased circumferential wall tension, normal tensile stress, and laminar flow/normal wall shear stress and a normotensive poststenotic segment associated with turbulent flow/low wall shear stress, normal circumferential wall tension and tensile stress in animals receiving a normal diet [15,17]. In addition to the observation of the effects of increased blood pressure levels and blood flow disturbances in aorta wall, the effects of cholesterol alone or associated with blood flow disturbances were desirable [18]. To this objective, a diet supplemented with 4% cholesterol, 1% cholic acid and 0.5% 2-thiouracil was offered, which is known to induce hypothyroidism and hypotension in rats [19]. The infradiaphragmatic aortic constriction in these animals produces a normotensive prestenotic segment associated with normal circumferential wall tension, normal tensile stress, laminar flow/normal wall shear stress and a normotensive poststenotic segment associated with turbulent flow/low wall shear stress, normal circumferential wall tension and normal tensile stress. This way, the effects of hypertension and laminar flow, hypercholesterolemia and laminar flow, turbulent blood flow and low wall shear stress, and turbulent blood flow and low wall shear stress associated with hypercholesterolemia could be evaluated.

**Fig. (1):**
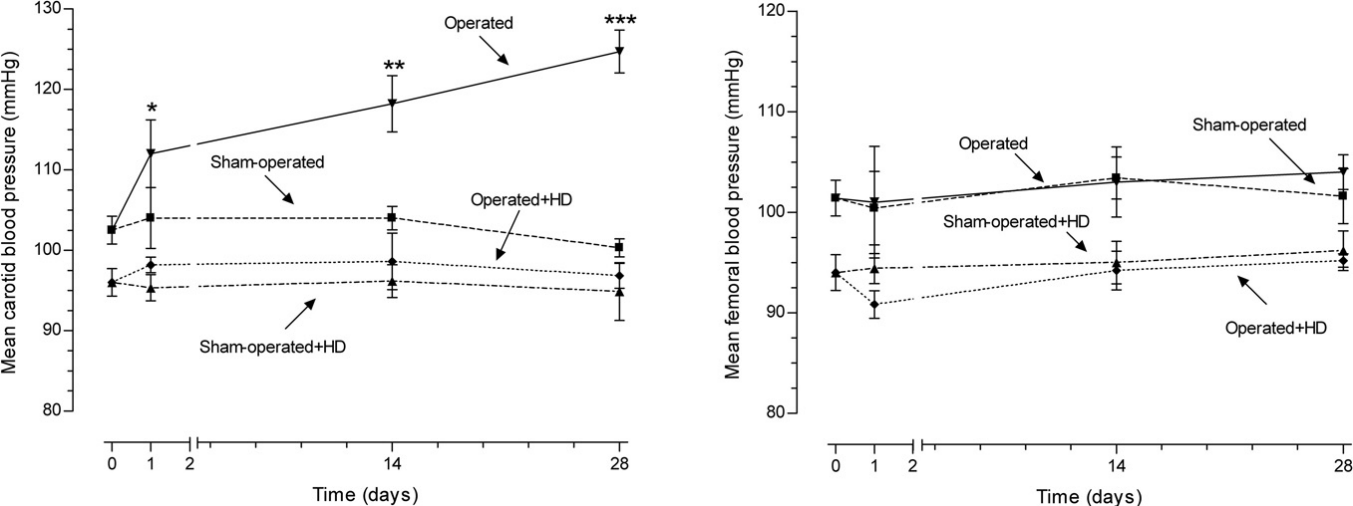
Mean carotid and femoral blood pressures in operated and sham-operated groups during the 28 day period of study. In the operated group, the increase in carotid pressure was progressive after constriction of abdominal aorta. The values of operated+HD group did not differ from sham-operated and sham-operated+HD values, although animals feeding HD have lower levels of arterial blood pressure. On the other hand, the femoral blood pressure was similar in all groups during the experiment.

The mean arterial blood pressure proximal and distal to the aortic constriction, carotid and femoral pressures, respectively, were obtained in time 0 (before surgery), 24 hours, 14 and 28 days after surgery (n=10 per day and per group). In the operated group, the increase in carotid pressure was progressive after constriction of abdominal aorta. After 24 h, the increase was 7.8% (p>0.05), after 14 days it was 13.2% (p<0.01), and at the end of the experiment, 24.3% (p<0.0001). The values of operated+HD group did not differ from sham-operated and sham-operated+HD values, although animals feeding HD have lower levels of arterial blood pressure. On the other hand, the femoral blood pressure was similar in all groups during the experiment (Fig. **1**).

## BLOOD FLOW ANALYSIS

**Fig. (2):**
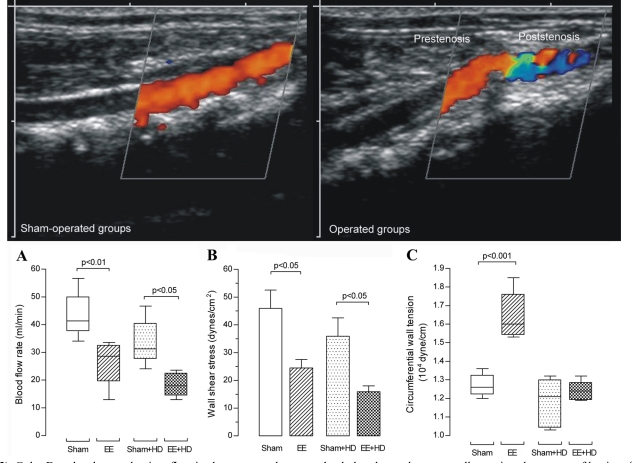
Color Doppler shows a laminar flow in sham-operated groups, the dark red near the aorta wall meaning slower rate of laminar flow. Color Doppler in operated groups demonstrates a preserved laminar flow in the prestenotic segment and a mixed red, blue, green and yellow characterizing turbulent flow in the poststenotic segment. (**A**) Blood flow rate (mL/min). Box and whisker graph shows the batches of data in sham-operated and operated groups at Day 28 of the experiment. (**B**) Wall shear stress mean values (dyne/cm2) in aortas from sham-operated and operated groups at Day 28 of the experiment. (**C**) Circumferential wall tension (104 dyne/cm) in aortas from sham-operated and operated

We performed aorta duplex ultrasonography and color Doppler in all groups after 28 days of the surgery of aorta coarctation (n=10 per group). Color Doppler showed a laminar flow in sham-operated and sham-operated+HD aorta and the orange-red color near the aorta wall meaning slower rate of laminar flow. Color Doppler in operated and operated+HD could demonstrate a preserved laminar flow appearing dark-orange to yellow in the prestenotic segment and a mixed of orange-red and blue in the poststenotic segment characterizing turbulent flow (Fig. **2**). Blood flow rate in the prestenotic segment was not different among all groups (data not shown). On the other hand, the values in the poststenotic segment were markedly lower in operated and operated+HD in comparison with sham-operated and sham-operated+HD, respectively (Fig. **2A**).

Wall shear stress (WSS) was calculated using the Poiseuille formula τ=4ηBFR/π(κr)^3^, where τ is WSS (dyne/cm^2^), η is the blood viscosity (0.03 poise), BFR is blood flow rate (ml/min), π is 3.14, κ is 1.25 (the shrinkage index, which is the ratio of artery diameter before and after plastic embedding) [20], and r is the arterial radius (cm) [21]. There was no difference among the values in the prestenotic segments in all groups (data not shown). The values in the poststenotic were markedly lower in operated and operated+HD in comparison with sham-operated and sham-operated+HD, respectively (Fig. **2B**).

## STRETCH EVALUATION *IN VIVO*

Mean circumferential wall tension (CWT) was calculated by Laplace Law according to the following formula: CWT=MPB x (ID/2), where mean circumferential wall tension is expressed in dyne/cm, MPB is mean blood pressure (dynes/cm^2^) and ID is internal diameter [14]. The ID of plastic embedded aorta was multiplied by 1.25 to correct the shrinkage after plastic embedding [20]. The mean CWT level in the prestenotic segment of operated group was markedly increased in comparison to values in sham-operated, sham-operated+HD and operated+HD groups (Fig. **2C**). The values in the poststenotic segments were no different among all groups (data not shown).

The medial thickening of 23% found in morphometrical analysis (data not shown) in the hypertensive prestenotic segment of operated group likely occurred in response to circumferential wall tension but not to circumferential deformation since diameter, perimeter, and luminal area of aortas in prestenotic segments were not different from those of sham-operated (data not shown). This contrasts with previous study showing that medial thickening occurred in response to circumferential deformation but not to circumferential tension in a vein-graft model in which tensions and deformation were discriminated using a band to narrow the carotid artery proximal to the vein-graft [12]. Ours results are consistent with an investigation using healthy humans demonstrating CWT positively correlated with carotid intima-media thickness [14]. It has been reported that intraluminal pressure regulates artery thickness through its effects on wall tension and blood flow regulates arterial lumen diameter through changes in wall shear stress [2, 12-14]. The increased wall thickness, mainly due to medial thickening, serves as a compensatory mechanism preventing increased arterial diameter, increased tensile stress and change of the wall shear stress. This medial thickening can be ascribed to increased expression of TGF-β in both endothelial and smooth muscle cells in the hypertensive segments compared with discrete expression of TGF-β in the corresponding segments in sham-operated aortas (immunohistochemistry staining, data not shown). TGF-β is a potent regulator of the cell cycle in many cells including vascular smooth muscle and endothelial cells [22]. This growth factor has been postulated to play an important, though largely undefined, role in vascular proliferative processes [23].

Tensile stress (TS) was computed as TS=CWT/IMT, where tensile stress is expressed in dyne/cm^2^, CWT is circumferential wall tension (dyne/cm) and IMT is intima-media thickness (cm) [14]. The mean TS values were not different in both prestenotic and poststenotic segments among all groups, sham-operated, operated, sham-opera-ted+HD and operated+HD (data not shown).

## MORPHOLOGICAL STUDY

After 28 days of surgery, the aortas were prepared for high resolution light microscopy and morphometric analysis (n=10 per group), immunohistochemistry (n=10 per group) and transmission electron microscopy (n=5 per group).

### High Resolution Light Microscopy

The gross examination revealed that both prestenotic and poststenotic segments were similar in all groups. The use of plastic embedding allowed 2 μm thick sections with good resolution of structural details.

**Fig. (3):**
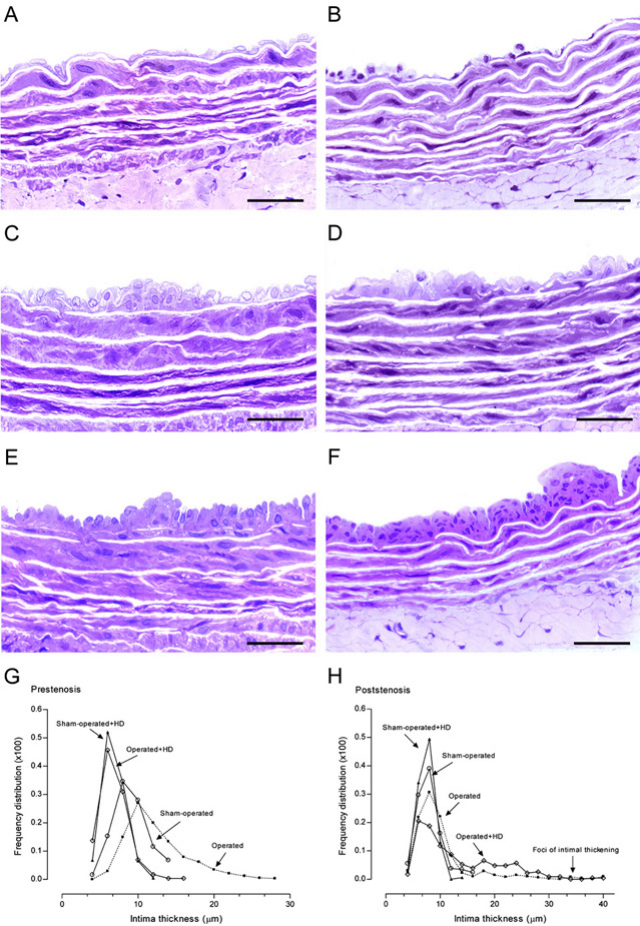
High resolution light microscopy. Representative views of the aortas from sham-operated (**A**), prestenosis (**C**), poststenosis (**E**), sham-operated+HD (**B**), prestenosis+HD (**D**) and poststenosis+HD (F). Panel **C**. Intimal thickening with enlarged endothelial cells and diffusely distributed neointimal plaques composed of smooth muscle cells and occasional mononuclear cells with collagen and elastic fibers surrounding them and medial thickening can be seen, contrasting with the delicate structure of the intima in the sham-operated group (**A**). Panel E. Intima appeared delicate quite similar to the intima in the sham-operated, except for focally distributed neointimal plaques similar to those observed in the prestenotic segment but many of them larger in size. Aortas from sham-operated+HD (Panel **B**) and operated+HD (Panel **D**) revealed diffusely distributed foci of small flat lesions corresponding microscopically to fatty streaks characterized by intimal foam cells accumulation, contrasting with the delicate structure of the intima in the sham-operated group. Panel F. Focally distributed incipient atherosclerotic lesions characterized by raised focal lesions within the intima composed of smooth muscle cells, mononuclear cells and extracellular matrix were seen in this segment. When the percentile frequency distribution of intima thickness in the prestenotic segment of all groups was plotted, it can be clearly seen the shift to the right of the values of operated group and in comparison with the values in corresponding segment in sham-operated animals (**G**). The percentile frequency distribution of intimal thickness in the poststenotic segment of sham-operated+HD, operated group and operated+HD was quite similar to that observed in corresponding areas of sham-operated group, except for the clear demonstrations of the occurrence of marked intimal thickening in the operated group and operated+HD (H). Scale bars, 40 µ m.

Aortas from operated group demonstrated in the hypertensive prestenotic segment changes characterized by intimal thickening with enlarged endothelial cells and diffusely distributed neointimal plaques composed of smooth muscle cells and occasional mononuclear cells with collagen and elastic fibers surrounding them and medial thickening (Fig. **3C**), contrasting with the delicate structure of the intima in the sham-operated group (Fig. **3A**). Aortas from sham-operated+HD (Fig. **3B**) and operated+HD (Fig. **3D**) revealed diffusely distributed foci of small flat lesions corresponding microscopically to fatty streaks characterized by intimal foam cells accumulation, contrasting with the delicate structure of the intima in the sham-operated group. When the percentile frequency distribution of intima thickness in the prestenotic segment of all groups was plotted, it can be clearly seen the shift to the right of the values of operated group and in comparison with the values in corresponding segment in sham-operated animals. The diffuse intimal thickening and diffusely neointimal plaques observed in the operated group and the small flat lesions observed in sham-operated+HD and operated+HD can be evidenced (Fig. **3G**).

In the poststenotic segment, aortas from operated group showed intima delicate quite similar to the intima in the sham-operated, except for focally distributed neointimal plaques similar to those observed in the prestenotic segment but many of them larger in size (Fig. **3E**). In operated+HD aortas, focally distributed incipient atherosclerotic lesions characterized by raised focal lesions within the intima composed of smooth muscle cells, mononuclear cells and extracellular matrix were seen in this segment (Fig. **3F**). The percentile frequency distribution of intimal thickness in the poststenotic segment of sham-operated+HD, operated group and operated+HD was quite similar to that observed in corresponding areas of sham-operated group, except for the clear demonstrations of the occurrence of marked intimal thickening in the operated group and operated+HD (Fig. **3H**).

### Transmission Electron Microscopy

The transmission electron microscopy of sham-operated aortas did not differ from that reported in the literature [15,17,24].

**Fig. (4):**
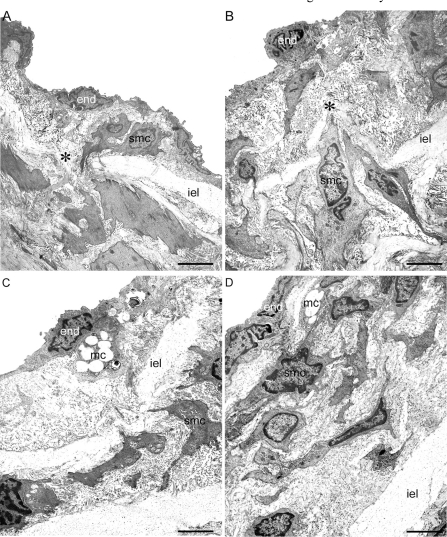
Transmission electron microscopy. Representative aspects of the prestenotic (**A**), poststenotic (**B**), sham-operated+HD and prestenotic+HD (**C**) and poststenotic+HD (**D**) segments. Panel A. The intima is thickened. The endothelial cells are heterogeneous, most of them with convoluted nuclei and cytoplasmic contours. Focal accumulation of smooth muscle cells could be seen in the expanded intimal layer. Migration of smooth muscle cells from the media into the intima (* ) through the fenestras of the internal elastic lamina can be also seen. Panel **B**. Marked intimal thickening composed of great number of smooth muscle cells surrounded by basement membrane-like material and collagen and elastic fibers can be seen. Migration of smooth muscle cells from the media into the intima (* ) is shown. Panel **C**. Represen-tative view of the small flat lesions corresponding to fatty streaks characterized by a few vacuolated mononuclear and smooth muscle cells accumulation. Panel **D**. Representative view of the poststenotic raised incipient atherosclerotic lesions composed of mononuclear and smooth muscle cells, many of them vacuolated, surrounded by extracellular matrix. End, endothelial cell; iel, internal elastic lamina; smc, smooth muscle cell; mc, mononuclear cell; * , migration of smooth muscle cell from the media to the intima. Scales bars, 2 µ m (**A**) and 3 µ m (**B, C, D**).

In the hypertensive prestenotic segment from operated group, the intima appeared diffusely expanded mainly due to enlarged endothelial cells showing irregular nuclear and cytoplasmic contours resting on a basement membrane-like material and delicate fibrocollagenous support tissue. Focally distributed discrete neointimal plaques could be also detected. They were composed of clusters of smooth muscle cells, randomly arranged, surrounded by basement membrane-like material, collagen and young elastic fibers (Fig. ****4A****). Migration of smooth muscle cells from the media into the intima through the fenestras could also be seen. The media remained intact, the smooth muscle cells appearing unaltered, retaining their orientation to the vessel. In the poststenotic segment the intima appeared comparable to that of corresponding segment of sham-operated, except for focally distributed neointimal plaques, larger than those observed in the prestenotic segments. The foci were similar to those observed in the prestenotic segment, but of larger size. No changes could be observed in the media (Fig. **4B**).

The aortas from sham-operated+HD and operated+HD appeared no different from that observed in sham-operated group, except for the focal lesions described above. The small flat lesions observed in sham-operated+HD and prestenotic segment from operated+HD (Fig. **4C**), corresponding to fatty streaks at the high resolution light microscopic study, were composed of mononuclear and smooth muscle cells with vacuolated cytoplasm surrounded by collagen matrix localized in the subendothelial space. In the poststenotic segment, the incipient atherosclerotic lesions at the high resolution light microscopic study were composed by vacuolated mononuclear cells and great number of smooth muscle cells, many of them vacuolated surrounded by collagen matrix (Fig. **4D**).

## IMMUNOHISTOCHEMISTRY

**Fig. (5):**
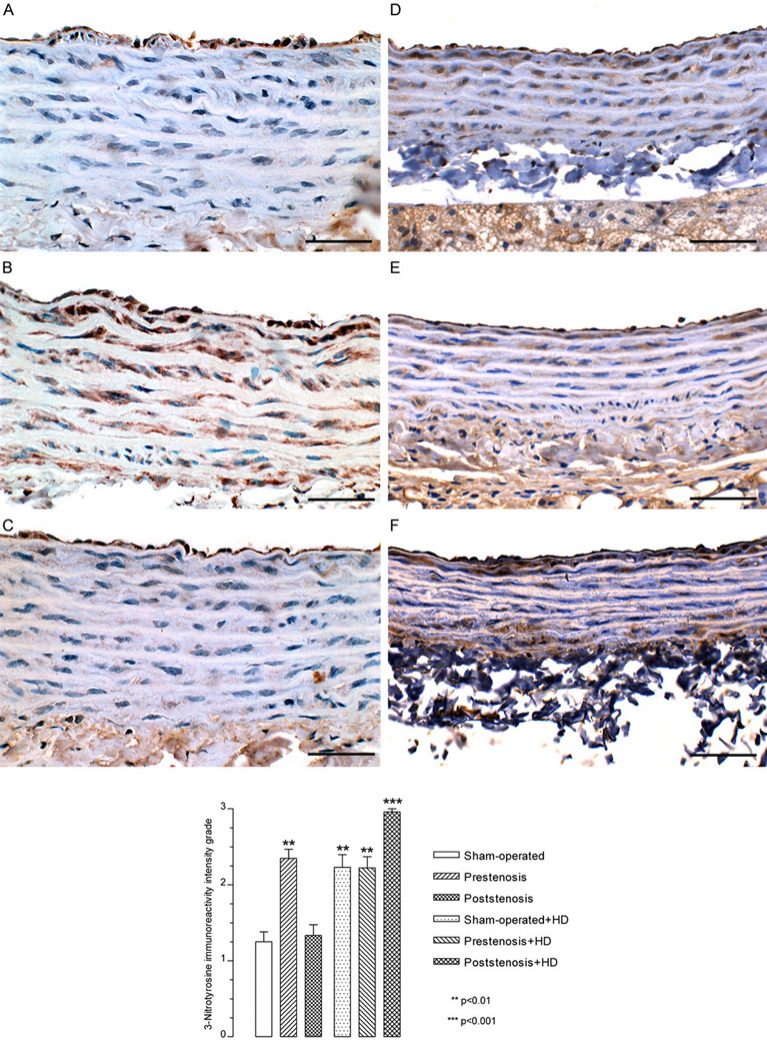
Immunohistochemistry. Representative views of the aortas from sham-operated rats (**A**), prestenotic (**B**) and poststenotic (**C**) segments from operated rats and sham-operated+HD (**D**), prestenotic (**E**) and poststenotic (**F**) segments from operated+HD. The graph represents the quali-quantitative evaluation of the 3-nitrotyrosine immunoreactivity grade. The analysis revealed an increased expression of 3-nitrotyrosine in endothelial cells and smooth muscle cells in the prestenotic segment from operated group, sham-operated+HD, prestenosis+HD and poststenosis+HD as compared to sham-operated group. Scale bars, 50 µ m.

The analysis revealed an increased expression of eNOS, iNOS (data not shown), and nitrotyrosine in endothelial cells and smooth muscle cells in the prestenotic segment from operated group (Fig. **5B**) as compared to sham-operated group (Fig. **5A**) and poststenotic segment (Fig. **5C**). It is likely that endothelial cell respond to hypertensive stress with augmentation of eNOS and iNOS expression as a compensatory mechanism aiming to increase production of nitric oxide (NO). In a similar model, marked upregulation of eNOS in the aortic segment proximal to coarctation compared with the corresponding segments in sham-operated control was found [25]. It has been suggested that NO production in large amounts by iNOS is a toxic-damaging agent [26], whereas eNOS is a protective enzyme [27]. The increased presence of NT in endothelial cells and, mainly, smooth muscle cells, as compared with the absence or discrete expression of NT in sham-operated indicates an increased production of NO and superoxide that interact to produce peroxynitrite, a powerful oxidant causing damage to multiple cell components including proteins [28]. The significant accumulation of NT, which is the footprint of NO oxidation/inactivation by reactive oxygen species [29], supports the supposition of endothelial dysfunction in the face of marked upregulation of eNOS and iNOS in this model, consistent with avid NO inactivation by oxidative stress in the affected vascular bed. This finding agrees with previous study showing marked increase in NT expression in the aortic segment proximal to constriction above the renal arteries [25]. An increased expression of eNOS in endothelial cells was detected in sham-operated+HD, prestenotic and poststenotic segment from operated+HD as compared with sham-operated group (data not shown). In addition, an increased expression of NT in endothelial cells and smooth muscle cells was detected in sham-operated+HD (Fig. **5D**), prestenotic (Fig. **5E**) and more markedly in the poststenotic segment (Fig. **5F**) from operated+HD as compared with sham-operated group. It has been demonstrated that in the presence of hypercholesterolemia, eNOS generates superoxide and the endothelium acts as a source of reactive oxygen species contributing to the early atherosclerotic process [30-33]. Hypercholesterolemia has been demonstrated to strikingly increase the expression of NT in endothelium, smooth muscle layers and adventitia of the aorta in rabbits [34]. The even more pronounced expression on NT in the poststenotic segments is very likely due to an additional factor, hemodynamic alterations. This assumption is supported by recent study on human coronary arteries showing that NT is present in arterial regions exposed to oscillatory shear stress (curvatures and bifurcations), but not in arterial regions exposed to pulsatile shear stress (straight segments) [35]. The accumulation of NT supports the supposition of endothelial cell dysfunction elicited by hypercholesterolemia. Although NT is considered an emergent inflammatory marker for atherosclerosis [36], the mechanism by which peroxynitrite formation contributes to atherogenesis remains uncertain.

## CONCLUSION

The aortas demonstrated distinct adaptive remodeling responses to different atherogenic stimuli: hypertension, hypercholesterolemia, turbulent blood flow/low wall shear stress and turbulent blood flow/low wall shear stress+hyper-cholesterolemia. The first is remodeling in the hypertensive prestenotic segment with increased circumferential wall tension associated with normal tensile stress and, laminar flow/normal wall shear stress characterized by enlarged heterogeneous endothelial cells, elongated in the direction of the blood flow, diffusely distributed neointimal plaques, appearing as discrete bulging toward the vascular lumen, and medial thickening. The second is remodeling in the normotensive prestenotic segment in the presence of hyper-cholesterolemia and oxidative stress with laminar flow, normal circumferential wall tension and tensile stress characterized by diffusely distributed foci of small flat lesions corresponding microscopically to fatty streaks. The third is remodeling in the normotensive poststenotic segment with turbulent blood flow/low wall shear stress and normal circumferential wall tension and tensile stress characterized by groups of endothelial cells with phenotypic alterations and focally distributed neointimal plaques, similar but many of them larger than those found in the prestenotic segments of operated group. The fourth is remodeling in the normotensive poststenotic segment in the presence of hypercholesterolemia and oxidative stress with turbulent blood flow/low wall shear stress and normal circumferential wall tension and tensile stress characterized by focally distributed incipient atherosclerotic lesions composed of smooth muscle cells, mononuclear cells and extracellular matrix. Our findings suggest that increased circumferential wall tension due to hypertension play a key role in the remodeling of the prestenotic segment of operated animals through biomechanical effects on oxidative stress and increased TGF-β expression. The remodeling observed in the presence of hypercholesterolemia could be initiated by oxidative stress that is involved in several processes of atherogenesis and this remodeling is more pronounced in the presence of turbulent blood flow/low wall shear stress. Further studies are needed to determine how the mechanical forces of turbulent flow/low shear stress are detected and transduced into chemical sign (s) by the cells of the artery walls and then converted into pathophysiologic relevant phenotypic changes.
